# The Nitrate Assimilatory Pathway in *Sinorhizobium meliloti*: Contribution to NO Production

**DOI:** 10.3389/fmicb.2019.01526

**Published:** 2019-07-03

**Authors:** Bryan Ruiz, Alexandre Le Scornet, Laurent Sauviac, Antoine Rémy, Claude Bruand, Eliane Meilhoc

**Affiliations:** Laboratoire des Interactions Plantes-Microorganismes (LIPM), INRA, CNRS, INSA, Université de Toulouse, Castanet-Tolosan, France

**Keywords:** nitric oxide, *Sinorhizobium meliloti*, denitrification, symbiosis, *Medicago truncatula*, nitrate assimilation

## Abstract

The interaction between rhizobia and their legume host plants culminates in the formation of specialized root organs called nodules in which differentiated endosymbiotic bacteria (bacteroids) fix atmospheric nitrogen to the benefit of the plant. Interestingly, nitric oxide (NO) has been detected at various steps of the rhizobium-legume symbiosis where it has been shown to play multifaceted roles. It is recognized that both bacterial and plant partners of the *Sinorhizobium meliloti*–*Medicago truncatula* symbiosis are involved in NO synthesis in nodules. *S. meliloti* can also produce NO from nitrate when living as free cells in the soil. *S. meliloti* does not possess any NO synthase gene in its genome. Instead, the denitrification pathway is often described as the main driver of NO production with nitrate as substrate. This pathway includes the periplasmic nitrate reductase (Nap) which reduces nitrate into nitrite, and the nitrite reductase (Nir) which reduces nitrite into NO. However, additional genes encoding putative nitrate and nitrite reductases (called *narB* and *nirB*, respectively) have been identified in the *S. meliloti* genome. Here we examined the conditions where these genes are expressed, investigated their involvement in nitrate assimilation and NO synthesis in culture and their potential role *in planta*. We found that *narB* and *nirB* are expressed under aerobic conditions in absence of ammonium in the medium and most likely belong to the nitrate assimilatory pathway. Even though these genes are clearly expressed in the fixation zone of legume root nodule, they do not play a crucial role in symbiosis. Our results support the hypothesis that in *S. meliloti*, denitrification remains the main enzymatic way to produce NO while the assimilatory pathway involving NarB and NirB participates indirectly to NO synthesis by cooperating with the denitrification pathway.

## Introduction

Rhizobia are Gram negative bacteria which are found as free-living organisms in the soil but also have the unique capacity to establish a nitrogen fixing endosymbiosis with legumes. Indeed rhizobia possess the capacity to reduce atmospheric nitrogen into ammonium to the benefit of their host plants thanks to their nitrogenase. As a consequence, in contrast to other plants, legumes can grow without addition of exogenous nitrogen (i.e., fertilizers) which is compatible with the development of sustainable agriculture practices.

Due to their agronomical and environmental interest, legume-rhizobium symbioses have been studied for many years and molecular determinants governing the recognition step between the two partners, the infection and the bacteroid differentiation process including nitrogen fixation, have been identified. Among those, nitric oxide (NO) has been detected at every step of the symbiotic interaction (Hichri et al., 2016b). NO is a diffusible and reactive gaseous molecule that plays a major signaling role in various processes in mammals. The importance of NO in plants emerged recently but it is now well described that it participates in numerous plant signaling pathways such as those controlling seed germination, root growth, flowering and stomatal closure (Domingos et al., 2015). Interestingly, it has also been shown that NO is involved in the hypersensitive response (HR) during the plant defense response against pathogen attack (Boccara et al., 2005).

Although the role of NO during the symbiotic interaction is not fully understood, it has been shown that NO can have a positive role during the infection steps, while it can inhibit the bacterial nitrogenase responsible for nitrogen fixation, and the plant glutamine synthetase involved in nitrogen assimilation (Trinchant and Rigaud, 1982; del Giudice et al., 2011; Melo et al., 2011). NO has also been shown to be a signal for nodule senescence (Cam et al., 2012). Hence, to maintain efficient infection and nitrogen fixation, the level of NO inside legume root nodules must be finely tuned. The NO level results from a balance between NO synthesis and consumption, two processes which rely on both partners (Hichri et al., 2016a). Indeed, both plant and bacterial hemoglobins have been shown to be involved in NO transformation or detoxification (Berger et al., 2018). In addition, the bacterial NO reductase Nor, catalyzing the reduction of NO into nitrous oxide (N_2_O) in the denitrification pathway is also involved in NO consumption in legume nodules.

Even though both plant and bacteroid participate in NO production, the relative contribution of each partner varies between different legume-rhizobium models (Sánchez et al., 2010; Horchani et al., 2011). In the symbiosis between *Bradyrhizobium japonicum* and soybean, bacteria account for about 90% of NO present in the nodules, while in the *S. meliloti–M. truncatula* symbiosis the bacteria produce about 35% of the amount of NO detected. Neither rhizobia nor legumes possess a gene that encodes a NO synthase of the type found in mammalian cells (Jeandroz et al., 2016). NO can originate from different routes in plants, the best characterized being the stepwise reduction of nitrate (Boscari et al., 2013; Astier et al., 2017). In *M. truncatula*, at least two out of the three nitrate reductase genes identified in the genome encode proteins that seem to be involved in NO synthesis (Berger et al., 2018). The second step of the reaction (i.e., production of NO from nitrite) could be catalyzed through the action of the mitochondrial electron transport chain in plants (Horchani et al., 2011).

**FIGURE 1:**
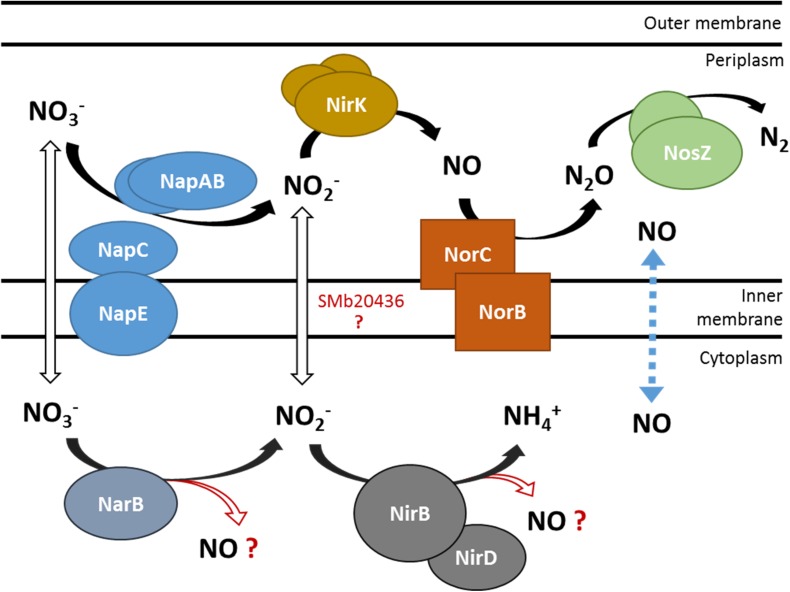
Schematic representation of nitrate utilization and NO production by the denitrification and putative NarB NirB pathways. NO_3_^–^ and NO_2_^–^ transporters are not identified yet in *S. meliloti*. The dotted line represents NO diffusion across the cytoplasmic membrane. Enzymes involved in the denitrification pathway: Nap (nitrate reductase), Nir (nitrite reductase), Nor (NO reductase), Nos (nitrous oxide reductase). SMb20436: *B. japonicum* NarK homolog, putative nitrite transporter. NarB, putative assimilatory nitrate reductase; NirBD, putative assimilatory nitrite reductase.

On the bacteroid side, the denitrification pathway, which has been well characterized in *B. japonicum* and *S. meliloti* (Figure 1) is the most likely source of NO, not only during the symbiotic interaction with legumes but also in soils (Torres et al., 2011, 2014). The regulation of this pathway has been well described in both organisms. In *S. meliloti*, the expression of *nap* genes is regulated in response to oxygen levels by the transcriptional regulator FixK, itself under the control of the two-component system FixLJ. The expression of the *nir* and *nor* genes is mainly regulated by the NO specific regulator NnrR, present in many rhizobia and, as a consequence, their expression depends upon the presence of NO (Meilhoc et al., 2010).

Recent work has shown that in *B. japonicum*, the nitrate assimilation pathway which includes a nitrate reductase NasC and an assimilatory nitrite reductase NirA, could also produce NO by a yet unknown mechanism (Cabrera et al., 2016). Luque-Almagro et al. (2011) examined a number of available genomes and suggested that the nitrate assimilation pathway in *S. meliloti* might include four genes (i.e., *nirB nirD narB cysG*). Assimilatory nitrate and nitrite reductase enzymatic activities were detected in *S. meliloti* cultures grown in presence of nitrate and reduced in the presence of ammonium (Kumar Halder and Chakrabartty, 2015). Interestingly NarB displays 53% identity and shares four main protein domains with NasC. NirBD is anticipated to be a siroheme-dependent assimilatory nitrite reductase that catalyses reduction of nitrite to ammonia and as such, shares the same function with NirA from *B. japonicum*. However, there is no synteny between both organisms regarding these genes. Indeed while both genes are located next to each other in the *S. meliloti* genome, in *B. japonicum* the genes that encode NasC and NirA are located at different loci. *cysG* (SMb20987) encodes a putative uroporphyrin-III C-methyltransferase involved in the synthesis of sirohaem, the nitrite reductase cofactor.

In this work we analyzed the expression profiles of *narB* and *nirB* genes in free-living conditions and *in planta*, and addressed the question whether they belong to the nitrate assimilatory pathway and are involved in NO synthesis. Finally, we examined different phenotypes of *M. truncatula* plants inoculated with a *S. meliloti* strain mutated in *narB* or *nirBD narB.*

## Materials and Methods

### Bacterial Strains and Growth Conditions

The bacterial strains and plasmids used are listed in Table 1.

**TABLE 1:** List of plasmids and strains used.

**Plasmids and strains**	**Description**	**References**
**Plasmids**		
pGEM-T	Cloning vector, Amp^r^	Promega
pGEM 5′UTR*nirB*	5′UTR*nirB* ligated with pGEM-T	This work
pGEM 5′UTR*narB*	5′UTR*narB* ligated with pGEM-T	This work
pGEM 3′UTR*narB*	3′UTR*narB* ligated with pGEM-T	This work
pJQ200mp19	Gene replacement vector, Gm^r^	Quandt and Hynes,1993
pJQ 5′3′UTR*narB*	5′3′UTRs*narB* ligated with pJQ	This work
pJQ 5′UTR*nir*B3′UTR*narB*	5′UTR*nir*B3′UTR*narB* ligated with pJQ	This work
***E. coli***		
DH5α	F-Φ80 l*acZ*ΔM15 Δ(*lacZY*α *argF*) U169 *recA1 endA1 hsdR17* (rk−, mk+) *phoA supE44 thi-1 gyrA96 relA1* λ-	Invitrogen
***S. meliloti***		
GMI11495 (CBT707)	*S. meliloti* 2011 Sm^R^	Pobigaylo et al.,2006
CBT612	CBT707 *napA*::Tn*5*, Sm^R^, Neo^R^	Pobigaylo et al.,2006
CBT614	CBT707 *nirK*::Tn*5*, Sm^R^, Neo^R^	Pobigaylo et al.,2006
CBT2473	CBT707 Δ*nirB nirD narB*, Sm^R^	This work
CBT2474	CBT707 Δ*narB*, Sm^R^	This work

*Sinorhizobium meliloti* strains were grown in Luria Bertani medium supplemented with 2.5 mM CaCl_2_ and 2.5 mM MgSO_4_ (LBMC). When necessary, antibiotics were added in the medium at the following concentrations: streptomycin (Sm) 100 or 300 μg/ml, neomycin (Neo) 100 μg/ml and gentamycin (Gm) 40 μg/ml. To test cell growth, gene expression or NO production, *S. meliloti* was grown in the exponential phase at an optical density at 600 nm of 0.2 (OD_600_ = 0.2) in Vincent Minimal Medium (VMM) with either NH_4_Cl (18.7 mM) or glutamate (10 mM) as nitrogen source, at 28°C (del Giudice et al., 2011). When needed, KNO_3_ (20 mM), or spermine NONOate (25 μM, from a 100 mM stock solution in NaOH 1 mM) were added to the culture. Microaerobic cultures were grown under a 2% oxygen atmosphere.

### Construction of *S. meliloti* Mutant Strains

All plasmid constructions were performed in *Escherichia coli* DH5α. The DNA sequences of oligonucleotide primers used for PCR amplification are shown in Table 2.

**TABLE 2:** List of oligonucleotides.

**Oligonucleotides**	**Sequences 5′-3′**	**Description**
OCB1557	GTCGACGCTGATCATTGCGA	fw *nirB5*’
OCB1558	GGATCCTTCAGTCATGTGGT	rev *nirB*5′
OCB1606	CGTCATCCTTGACCAGGGTC	fw *nirBDnarB* deletion screening
OCB1567	GTCGACGACAAGCACGAGTT	fw *narB*5′
OCB1568	GGATCCTCTTATTCCGCCGC	rev *narB*5′
OCB1569	GGATCCATCTGGCCCAAAAG	fw *narB*3′
OCB1570	GAGCTCCGATCCGAACTGGA	rev *narB*3′
OCB1611	CCTATTTCGACCGCTTCGTC	fw *narB* deletion screening
OCB1612	CGGATCGCCTGTGCCAGG	rev *narB* and *nirBDnarB* deletion screening
OCB1607	CTGCCGAAATTGGCGGGATG	*nirB* rev
OCB1587	TTCTTCCGGCTCTTCGAAAC	qRTPCR *narB* fw
OCB1588	CGAGATGGCAGTTGATGATG	qRTPCR *narB* rev
OCB1583	ATGTCATGCCGACACTGATG	qRTPCR *nirB* fw
OCB1584	CTTTGTGACGACCTTGATGC	qRTPCR *nirB* rev
OCB1638	GTCATCGACCTCACCCATTT	qRTPCR SMc01171 fw
OCB1639	TCTGCAGCAAGAACCACTTG	qRTPCR SMc01171 rev
OCB1914	TCGGCGTCAACCAGATCACTGC	qRTPCR *narB* fw
OCB1915	AATGCTTCTGCATCGGATTGCTCG	qRTPCR *cysG* rev

To construct the *narB* mutant, the 5′ and 3′ flanking regions of *narB* were amplified by PCR by using genomic DNA of the strain GMI11495 (CBT707) as template and the oligonucleotides OCB1567/OCB1568 to amplify the 5′ flanking region (413 bp) and OCB1569/OCB1570 to amplify the 3′ flanking region (413 bp). The PCR fragments were then ligated with pGEM-T yielding plasmids pGEM 5′UTR*narB* and pGEM 3′UTR*narB*, respectively. The cloned *S. meliloti* regions were verified by DNA sequencing. The plasmids were digested with either *Bam*HI/*Sal*I or BamHI*/Sac*I to isolate the 5′ and 3′ UTR, respectively. The UTRs were ligated with pJQ200mp19 digested with *Sal*I/*Sac*I yielding pJQ 5′3 ′UTR*narB*.

pJQ 5′3′UTR*narB* was introduced into *S. meliloti* GMI11495 (CBT707) by electroporation to yield the strain CBT2474 (Ferri et al., 2010; Dupuy et al., 2017). For this, first a single crossing over genomic integration was selected by Gm resistance. The resulting strain was then grown in the absence of antibiotics and cells having lost the plasmid following a second recombination event were selected by plating on LBMC medium supplemented with 5% sucrose as the plasmid carries the *sacB* gene which is lethal for *S. meliloti* in the presence of sucrose. A Gm sensitive clone that grew on sucrose containing medium was selected and the deletion was verified by PCR using the primers OCB1611/OCB1612 and by sequencing the product.

The whole *nirBD–narB* region was deleted by constructing a pJQ200mp19 plasmid containing the 5′*nirB* flanking region and the 3′*narB* flanking region. The 5′ flanking region of *nirB* was amplified by PCR by using genomic DNA of the strain GMI11495 (CBT707) as template and the oligonucleotides OCB1557/OCB1558 as primers (fragment size 423 bp). The PCR fragment was then ligated with pGEM-T yielding plasmid pGEM 5′UTR*nirB*. The cloned *S. meliloti* region was verified by DNA sequencing. The plasmids pGEM 5′UTR*nirB* and pGEM 3′UTR*narB* were digested with either *Bam*HI/*SalI* or BamHI*/Sac*I to obtain the 5′ and 3′ UTR, respectively. The UTRs were ligated with pJQ200mp19 digested with *Sal*I/ *Sac*I yielding pJQ 5′UTR*nir*B3′UTR*narB*. The plasmid was introduced into *S. meliloti* GMI11495 (CBT707) by electroporation to give the strains CBT2473. The deletion was verified by PCR using the primers OCB1606/OCB1612 and by sequencing the product.

### Measurement of NO Production in *S. meliloti* Cultures

*Sinorhizobium meliloti* strains were grown exponentially at 28°C in VMM containing either NH_4_Cl or glutamate (OD_600_ = 0.2–0.3). To remove NH_4_Cl or glutamate from the medium, cells were collected by centrifugation (8000 × *g*, 10 min), washed once with sterile water, resuspended in VMM without NH_4_Cl or glutamate. When needed, KNO_3_ (20 mM) was added to the cultures. Cultures were then either kept at 28°C under agitation (aerobic conditions) or left without agitation under 2% oxygen atmosphere (microaerobic conditions) for 1.5 h. The NO produced by bacteria and released in the culture medium was quantified using the fluorescent non-permeable probe DAF-2 (Sigma-Aldrich). To measure NO level, 1 ml of culture was transferred to a tube containing DAF-2 (1 μl of a 5 mM solution in DMSO). Tubes were then kept for 1 h at 20°C in the dark. 100 μl of the suspension were then transferred to a 96 well plate (Greiner, dark bottom). Fluorescence was measured using a microplate spectrofluorimeter (Fluostar Omega, BMG Labtech, Champigny sur Marne, France) (excitation wavelength 485 nm/emission wavelength 520 nm). NO produced is expressed as fluorescence/OD_600_ (measured just before incubation with DAF-2). Different concentrations of the NO donor, spermineNONOate, were tested under the same conditions to verify that the fluorescence obtained was proportional to the NO amount. In addition, two other controls were performed to test the method specificity (data not shown): (1) the fluorescence signal was drastically increased when using an *hmp S. meliloti* mutant strain affected in NO degradation, (2) the fluorescence was reduced when using an *S. meliloti* strain overexpressing *hmp* (Cam et al., 2012). The experiment was repeated four times, with two technical repeats for each biological repeat.

### Nitrite Determination in the Culture Medium

Cultures were grown as described above. For every culture sample collected to measure NO production, a second 1 ml aliquot was withdrawn to assay nitrite concentration in the medium. For this, the sample was centrifuged (10000 *g*, 5 min) and 100 μl of supernatant were incubated for 30 min (room temperature) with the Griess reagent (Nicholas and Nason, 1957). Absorbance was measured at 540 nm. NO_2_^–^ concentration was calculated from a calibration curve using NaNO_2_ (from 0 to 50 μM). The experiment was repeated five times, with two technical repeats for each biological repeat.

### RNA Extraction

Strains were grown exponentially at 28°C in VMM. At an OD_600_ between 0.2 and 0.3, Spermine NONOate (25 μM) or KNO_3_ (20 mM) was added to the culture. Cultures were incubated at 28°C either under agitation in aerobic conditions or under 2% oxygen atmosphere without agitation. 1.5 and 3 h later, cells (20 ml) were collected by filtration, immediately frozen in liquid nitrogen and stored at −80°C until RNA extraction. When needed, NH_4_Cl was removed as described before.

RNA was prepared from the collected samples by incubating filters for 20 min at 65°C in lysis buffer (SDS 1.4%, EDTA 4 mM, Proteinase K 40 μg/ml). Lysates were then incubated at 4°C (10 min) in presence of NaCl (1.7 M) and centrifuged. Nucleic acids were precipitated from the supernatant in the presence of isopropanol (4°C, 1 h), washed with ethanol 70%, dried and resuspended in DEPC-treated water (100 μg/μl). RNA was purified using the RNeasy kit (Qiagen) followed by DNAse treatment (TURBO DNA-free kit, Invitrogen) (37°C, 1 h). Sample concentration and purity were measured with a nanodrop (Nanodrop ND-1000, ThermoFisher Scientific).

### qRT-PCR Analysis

Reverse transcription was performed using Superscript II (Invitrogen) and random hexamers as primers. RNA samples isolated from at least three independent cultures were tested for each condition. Real time RT-PCR tests were run on a Light Cycler 480 (Roche) using the SYBR Green I Master kit (Roche) according to the manufacturer instructions. A calibration curve was established for each gene with known amounts of *S. meliloti* genomic DNA. The *S. meliloti* gene SMc01171 was used as a reference for normalization as its expression was constant in all conditions tested. Primers used for expression analyses of each gene are indicated in Table 2.

The cDNA generated were also used for amplification of putative intergenic regions between *nirBD* and *narB* using primers OCB1611/OCB1607 and between *narB* and *cysG* using primers OCB1914/OCB1915. In negative controls, reverse transcriptase was omitted while in positive controls PCR was performed with *S. meliloti* genomic DNA as template.

### Plant Assays

Seeds of *M. truncatula* cv jemalong A17 were surface sterilized, germinated on agar plates and allowed to grow on nitrogen-free Fahreus medium in test tubes during 2 days (Garcia et al., 2006). Series of plants were inoculated with either the wild type or the mutant strains (100 μl per plant of a resuspension in sterile water at OD_600_ = 0.001) or with 100 μl water as a control. Plants were grown in a culture room (22°C) with day and night periods of 16 and 8 h, respectively. Dry weights of the plant shoots were measured 4 and 5 weeks post-inoculation.

Nodules were macroscopically estimated as senescent when a green color was visible on a significant part of the nodule volume. The proportion of senescent nodules for each plant was calculed by dividing the number of senescent nodules by the total number of nodules (expressed in %).

The average number of nodules per plant, the percentage of senescent nodules and the dry weight of shoots were calculated from 19 to 27 plants (total number of plants obtained from three independent series).

Nitrogenase activity was determined by the acetylene reduction assay (Hardy et al., 1968), using plants grown in test tubes fitted with rubber stoppers. 1 ml of acetylene was added to each tube. Plants were then incubated in the growth chamber for 3 h. One ml of gas was taken from each tube and analyzed for ethylene content, using a gas chromograph equipped with a hydrogen flame ionization detector (7820A Agilent). The amount of ethylene produced by each plant was assessed by measuring the ethylene peak area and comparing to a standard (ethylene). Nitrogenase activity was measured on 7–9 plants for each genotype and each time point.

## Results

### Expression of *narB* and *nirB* Genes in *S. meliloti*

Even though *narB* and *nirB* are assumed to be part of the nitrate assimilation pathway in *S. meliloti* no experimental data were available (Luque-Almagro et al., 2011). Ferroni and colleagues measured nitrate reductase total activity in *S. meliloti* cultures grown under aerobic and microaerobic conditions in the presence of nitrite and/or nitrate (Ferroni et al., 2011). These authors suggested that nitrate reductase activity in aerobic conditions could be associated with assimilatory ammonification, in line with the work of (Sekiguchi and Maruyama, 1988). A more recent study in *S. meliloti* showed that a nitrate assimilatory enzyme activity, estimated through detection of nitrite in the medium, was observed when nitrate was in the medium and that the generation of nitrite was drastically reduced when ammonium was also present (Kumar Halder and Chakrabartty, 2015). No link has been made so far between assimilatory nitrate/nitrite reductase activities and the genes encoding these functions.

To gain insight into the conditions of expression of *narB* and *nirB* we analyzed gene expression in different culture conditions by using qRT-PCR.

**FIGURE 2:**
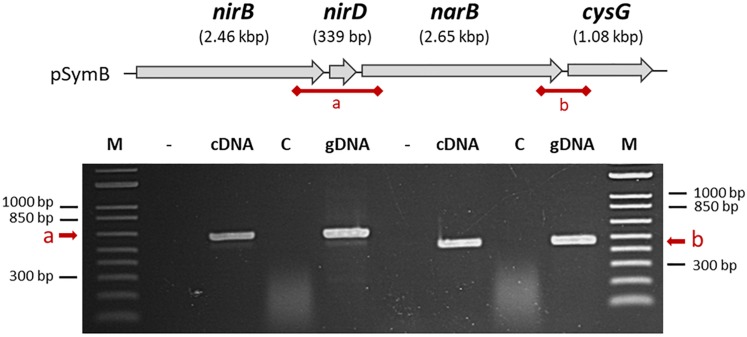
Organization of *nirB*, *nirD*, and *narB* genes on the *S. meliloti* genome. *nirB, nirD*, *narB*, and *cysG* genes are located on the *S. meliloti* megaplasmid pSymB as indicated at the top of the figure. The putative intergenic regions which are tested by PCR in the bottom part of the Figure are called (a) and (b). Total RNA isolated from cells grown in aerobic conditions in VMM without NH_4_Cl in the presence of nitrate served as template for cDNA synthesis in the presence (cDNA) or in absence of reverse transcriptase enzyme (control, C). Genomic DNA was used as a positive control for PCR amplifications. The first lane (–) is a negative control without DNA.

First, to investigate the transcriptional architecture of the region, PCR experiments were performed on cDNA to detect whether a transcript spanned the intergenic regions shown in Figure 2 between *nirBD* and *narB* (a) or between *narB* and *cysG* (b). PCR generated a 600 bp DNA fragment for the region labelled as (a) indicating that *narB*, *nirB, and nirD* could be in a single transcriptional unit. Similarly, a 473 bp fragment was obtained for region (b) showing that *cysG*, located downstream of *narB* could also be transcribed with it.

**FIGURE 3:**
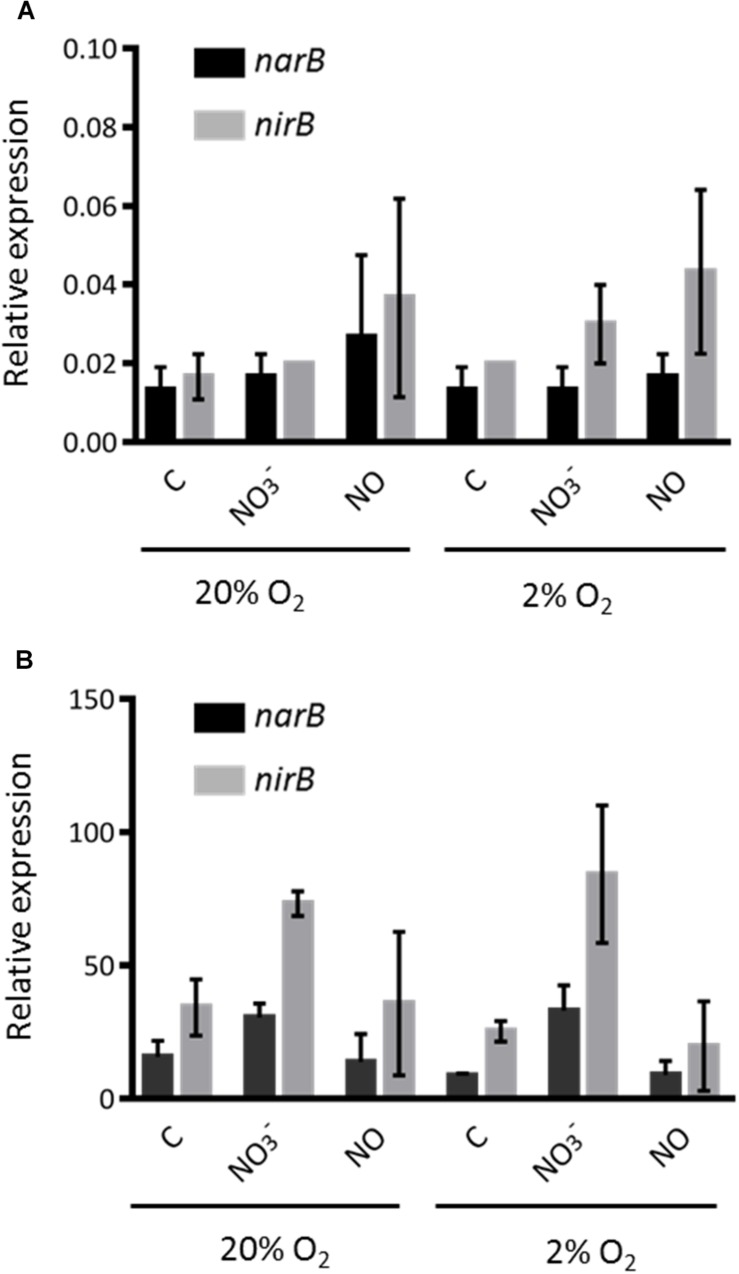
Expression level of *nirB* and *narB* genes. **(A)** *S. meliloti* WT strain was grown to exponential phase in Vincent minimal medium (with NH_4_Cl) (OD_600_ = 0.2–0.3). Cells were then exposed (1.5 h) to a NO donor (25 μM spermineNONOate) or nitrate (20 mM KNO_3_) in aerobic (20% O_2_) or microaerobic (2% O_2_) conditions. Cells were recovered by filtration and frozen in liquid nitogen. RNA was extracted and transcript levels of *narB* (black bars) and *nirB* (gray bars) were determined by qRT-PCR. Transcript levels were normalized by using *SMc01171* as a reference gene. **(B)** *S. meliloti* WT strain was grown as in **(A)** except that NH_4_Cl was removed from the medium by centrifugating cells and resuspending them in Vincent minimal medium without NH_4_Cl before exposing cells to the different conditions tested. All values shown are the means and standard errors of the mean of data from three independent experiments (two technical repeats for each experiment).

*narB* and *nirB* expression was first analyzed in conditions in which denitrification genes are known to be expressed. Expression was analyzed 1.5 and 3 h after addition of either nitrate or NO donor. A decrease in gene expression level was observed at 3 h (data not shown); hence, only the set of results obtained at 1.5 h is shown in Figure 3A. We first observed that the expression of both genes were at comparable levels, confirming that they share the same regulation, as expected if they were part of the same operon. The expression level was low whether cells were grown in aerobic (20% O_2_) or microaerobic conditions (2% O_2_). Expression was not significantly affected by addition of nitrate or a NO donor in the medium. These results are in agreement with previous microarray data which did not identify these genes as being regulated by low oxygen or NO (Bobik et al., 2006; Meilhoc et al., 2010). *napA* and *nirK* expression was assessed in the same set of experiments. *napA* expression level was low, with about the same order of magnitude as *narB* and *nirB*. *nirK* was found to be expressed at a much higher level, especially when NO was added to the medium (in either aerobic or microaerobic conditions) in agreement with previous results (Meilhoc et al., 2010) (data not shown). In a second set of experiments we removed NH_4_Cl from the culture medium as ammonium is known to repress assimilatory nitrate reductase activity. The results obtained are shown in Figure 3B. Strikingly, the expression of *narB* and *nirB* was about 1000-fold higher when ammonium was removed from the medium, indicating that *narB* and *nirB* gene expression was indeed inhibited by ammonium. Both *narB* and *nirB* displayed similar expression patterns and their expression was comparable whether cells were incubated in aerobic or microaerobic conditions. NO did not influence significantly *narB* and *nirB* gene expression and nitrate only slightly induced the expression level of both genes. *napA* and *nirK* expression levels were not significantly modified when NH_4_Cl was removed from the medium (data not shown).

### Growth of *S. meliloti* Strains in Presence of Nitrate

**FIGURE 4:**
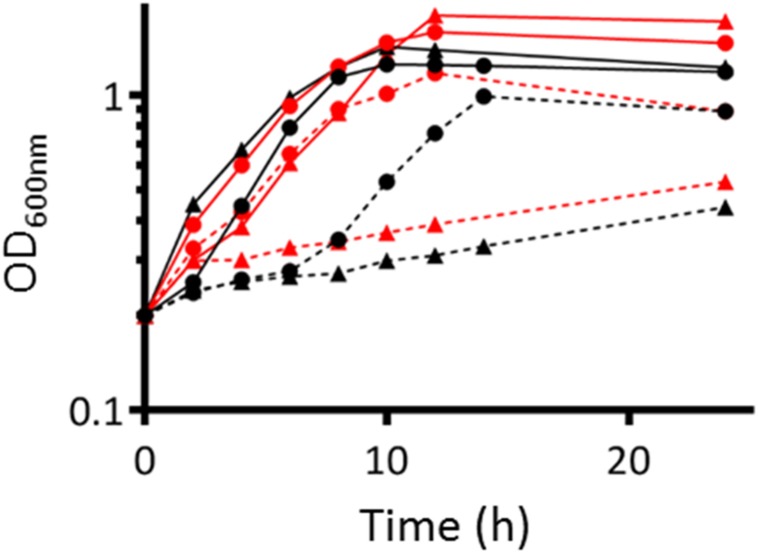
Growth of WT and *nirBD narB S. meliloti* strains. WT (●) or *nirBD narB* (▲) strain were grown to exponential phase (OD_600_= 0.2–0.3) in Vincent Minimal Medium (VMM) containing either glutamate (red lines) or NH_4_Cl (black lines) as the sole nitrogen source. At time zero, cells grown in VMM with glutamate or NH_4_Cl were collected, centrifuged, washed once by centrifugation, and resuspended at the same OD_600_ in VMM containing only KNO_3_ (dotted line), or a combination of KNO_3_ and glutamate (red solid line) or NH_4_Cl (black solid line). OD_600_ was measured over a 24 h period. A representative experiment is shown on the figure.

In order to test whether NarB and NirBD are involved in nitrate assimilation, we grew both the WT and *nirBDnarB* strains in aerobic conditions, in Vincent minimal medium (VMM) containing either nitrate as the sole nitrogen source or a combination NH_4_Cl/nitrate or glutamate/nitrate. The results are shown in Figure 4. Growth of the WT strain was comparable whether the nitrogen source in the medium was NH_4_Cl or glutamate. When glutamate was removed from the medium and replaced by nitrate the WT strain also displayed similar growth kinetics. Interestingly, when NH_4_Cl was removed from the culture medium before adding nitrate as the sole nitrogen source, WT cells displayed a 6 h lag phase before resuming growth. This could be explained by a previous repression of *narB nirB* gene expression by NH_4_Cl which could limit nitrate assimilation. The *narB nirBD* mutant growth was similar to that of the WT when glutamate or NH_4_Cl was present in the medium. Remarkably, the strain lacking NarB and NirBD was barely able to grow when nitrate was the sole N source present in the medium. These results suggest that *narB nirBD* genes are involved in nitrate assimilation.

### Production of NO From the NarB NirBD Pathway

**FIGURE 5:**
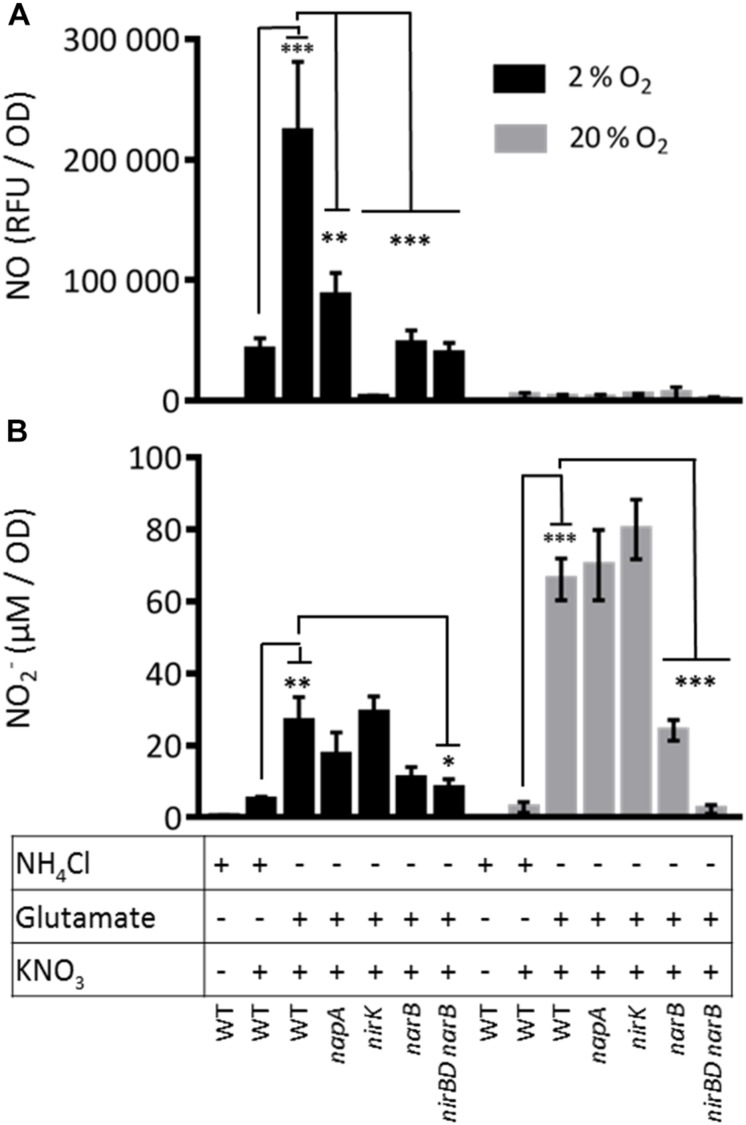
Nitric oxide and nitrite production by *S. meliloti* WT and mutant strains. *S. meliloti* strains were grown exponentially at 28°C in VMM containing either NH_4_Cl or glutamate (OD_600_= 0.2–0.3). To remove NH_4_Cl or glutamate from the medium, cells were collected by centrifugation and resuspended in VMM without NH_4_Cl or glutamate. When needed, KNO_3_ (20 mM) was added to the cultures. Cultures were either kept at 28°C under agitation (aerobic conditions, gray bars) or left without agitation under 2% oxygen atmosphere (microaerobic conditions, dark bars) for 1.5 h. Samples were collected to measure: **(A)** NO production : NO produced by the bacteria and released in the culture medium was quantified using the fluorescent non-permeable probe DAF-2. Fluorescence was measured using a microplate spectrofluorimeter (excitation wavelength 485 nm/emission wavelength 520 nm). NO produced is expressed as relative fluorescence unit (RFU)/OD_600 nm_. **(B)** Nitrite concentration in the medium: 100 μl of culture medium was incubated for 30 min with the Griess reagent. Absorbance was measured at 540 nm. NO_2_^–^ concentration was calculated from a calibration curve using NaNO_2_ (from 0 to 50 μM). The means and standard errors of the mean obtained from 4 (NO production) or 5 (Nitrite concentration) independent experiments are presented on the Figure. Statistical analysis was performed by means of a one-way ANOVA followed by a Bonferroni statistical test. ^*^, ^∗∗^, and ^∗∗∗^ indicate significant difference (*P* < 0.05, *P* < 0.01, and *P* < 0.001, respectively) when compared with the wild type strain (WT).

The only enzymatic source of NO described so far in *S. meliloti* is the denitrification pathway. Indeed Horchani and colleagues used *napA* and *nirK* mutants to demonstrate that about 35% of NO inside *M. truncatula* root nodules was produced by the bacteria (Horchani et al., 2011). To confirm these results we tested the involvement of *napA* and *nirK* in the production of NO in *S. meliloti* cultures grown in microaerobic conditions (oxygen 2%). We first examined the production of NO in the presence of nitrate in a WT strain grown in Vincent minimal medium (VMM) containing NH_4_Cl or glutamate (Figure 5A). NO was produced in both cases and to a higher extent (fivefold) when glutamate was used as a nitrogen source. NO was not detectable in absence of nitrate. We found that a *napA* mutant still produced about 39% of the amount of NO measured in the WT strain suggesting that either there might be an independent way of producing NO or an alternative way to produce nitrite.

To test the involvement of *narB* and *nirB* in NO synthesis, we constructed a *narB* deletion mutant and a mutant deleted for both *nirB* and *narB*. It has to be noted that this deletion also encompasses *nirD*, a gene located between *nirB* and *narB*. *nirD* is predicted to encode a small protein (112 amino acids) displaying homology with a nitrite reductase small subunit probably involved in the electron transfer to NirB. As *narB* and *nirB* expression is repressed in the presence of ammonium, we grew these strains in VMM medium containing glutamate and nitrate.

Under these conditions, the *narB* and *nirBDnarB* mutants produced only 21 and 17% of the NO produced by the WT strain, respectively. A similar decrease was observed when NH_4_Cl was present in the culture medium, conditions where *narB* and *nirB* were poorly expressed. In these experiments, NO was measured with a different method (chemiluminescence, data not shown). These data show that NarB and NirB have a role to play in NO production. Strikingly, the amount of NO dropped to almost zero in a *nirK* mutant showing that NO is produced by reduction of nitrite and indicating that the NarB/NirB involvement is likely due to the generation of nitrite by NarB. In this context it is interesting to note that NO production in the WT grown in the minus ammonium conditions where *nirBDnarB* were fully expressed, was increased by a factor of about 4 compared to the condition where ammonium was present. This observation also supports the idea that NarB NirBD promote NO production by generating nitrite to be used by the denitrification pathway.

In order to test whether NarB NirBD are involved in NO synthesis independently from the denitrification pathway, we tested NO production from the same strains grown in the same media as described above in aerobic conditions (Figure 5A). Under these conditions, the denitrification pathway and especially the NirK nitrite reductase is not active. The results clearly show that NO was not produced in significant amount in the WT strain growing in aerobic conditions in VMM with glutamate and nitrate. These results strongly suggest that NarB NirBD only participates in NO production when denitrification is active. To verify whether NarB NirBD were active under aerobic conditions, we quantified the amount of nitrite produced by the different strains in the same conditions as for the NO production assay. The results are shown in Figure 5B. Interestingly, in aerobic conditions, in the presence of nitrate and ammonium, nitrite was barely detectable in the medium while in the presence of glutamate a much higher level (20-fold) of nitrite was measured, indicating that NarB was functional in these conditions. Nitrite production by the *napA* and *nirK* mutants was not significantly different from that of the WT strain. Nitrite production in the *narB* and *nirBD narB* strains was reduced by 60 and 95%, respectively as compared to the WT. These data show that the NarB- NirBD pathway was active in aerobic conditions even though no production of nitric oxide could be measured. When the same experiment was performed in microaerobic conditions (Figure 5B) the level of nitrite was much lower, perhaps due to nitrite reductase activity in the denitrification pathway.

Nitrite was only produced when nitrate was present in the medium. The amount of nitrite produced by the WT strain was higher when the strain was grown in presence of glutamate as compared to ammonium suggesting that NarB could participate to nitrite production in these conditions. Accordingly, the nitrite production was reduced by a factor of 2.5 and 3 in the *narB* or *nirBD narB* mutant strains, respectively. Altogether these results show that in conditions where only *narB* and *nirB* are expressed there is a production of nitrite but not NO.

### Role of NarB and NirB *in planta*

**FIGURE 6:**
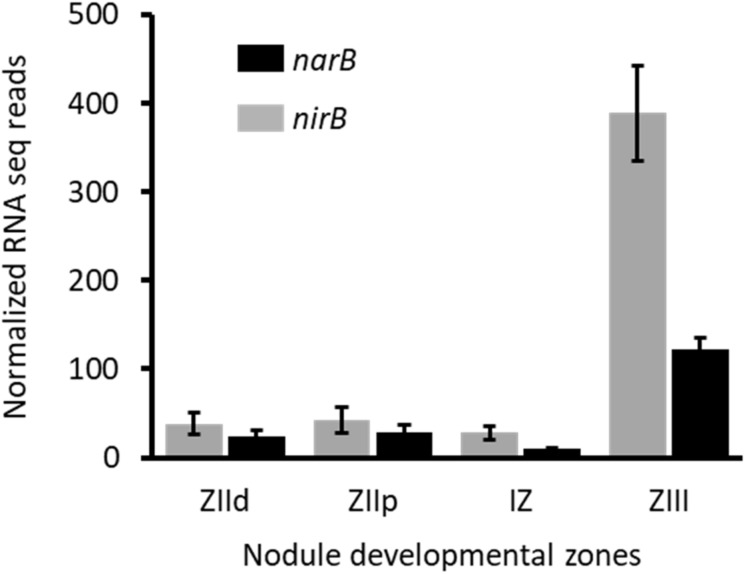
Expression level of *nirB* and *narB* genes in nodules. Data shown on this figure are extracted from the work of Roux et al. (2014) (https://iant.toulouse.inra.fr/symbimics). They represent the RNA–seq analysis of bacterial transcripts of *narB* (black) and *nirB* (gray) obtained from laser–dissected *M. truncatula* nodules occupied by a WT *S. meliloti* strain. ZIId, infection zone, distal part; ZIIp, infection zone, proximal part; IZ, interzone; ZIII, nitrogen fixation zone.

To assess *narB* and *nirB* gene expression in symbiosis, we analyzed transcriptomic data generated by Roux and colleagues (Roux et al., 2014). *M. truncatula* forms indeterminate nodules in which all the developmental stages can be seen in a mature nodule. These different zones are: the meristem (Zone I), the infection zone (Zone II distal and proximal) where bacteria are released in the plant cells, the Interzone (IZ) between Zones II and III, and the nitrogen fixing zone (Zone III). Roux et al. (2014) isolated each zone by laser microdissection and the plant and bacterial RNA they contained were extracted and sequenced. The zone-specific expression of *narB* and *nirB* genes is reported in Figure 6. The strongest expression of *narB* and *nirB* was observed in the nitrogen fixing zone (ZIII). *nirK* was also expressed in ZIII but to a lesser extent than *nirB* (not shown). Therefore *narB* and *nirB* are expressed in bacteroids contained in plant nodules and particularly in the fixation zone.

**FIGURE 7:**
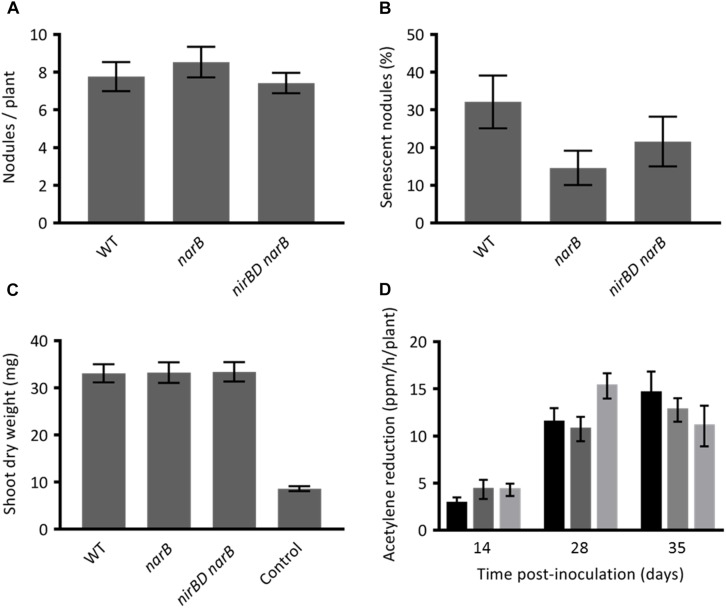
Phenotypes of *Medicago truncatula* inoculated with *narB* or *nirBD narB* mutants. *M. truncatula* plantlets were inoculated with *S. meliloti* WT or mutant strains. **(A)** The number of nodules per plant (19 to 27 plants were tested). **(B)** Percentage of senescent nodules. A nodule was estimated senescent when a green color was visible on a significant surface of the nodule (19–27 plants were tested). **(C)** The dry weight of the aerial part of the plant (18–20 plants were tested). These three parameters were tested 5 weeks post-inoculation. **(D)** The nitrogen fixation activity was assessed by using the acetylene reduction assay (ARA). ARA was performed at 14, 28, and 35 dpi on whole plants inoculated with the WT strain (black bars), *narB* mutant (dark gray bars) or *nirBD narB* mutant (light gray bars). For each strain and time point 7–9 plants were tested. All values are the mean ± standard error of the mean (SEM).

As these genes are more highly and specifically expressed in the nitrogen fixation zone we hypothesized that they could have a role to play in the symbiotic interaction. To assess the role of these genes *in planta* we inoculated series of *M. truncatula* plantlets with the WT strain or with *narB* and *nirBD narB* mutants and analyzed different plant phenotypes such as the number of root nodules (Figure 7A), appearance of senescent nodules (Figure 7B), shoot dry weight (Figure 7C), and nitrogen fixation (Figure 7D). The average number of nodules per plant was identical when these plants were inoculated with the wild type or the mutant strains. Five weeks post-inoculation of the plants with the WT strain, 33% of the nodules were senescent. When plants were inoculated with the mutant and WT strains the proportion of senescent nodules was comparable though sligthly lower in the mutants. Nitrogen fixation measured 2, 4, or 5 weeks post-inoculation was more or less similar for all strains. Dry weight of shoots measured 5 weeks post-inoculation was also similar. Hence *narB* or *nirBD narB* deletions do not lead to substantial effects on various aspects of plant fitness during symbiosis.

## Discussion

### NarB and NirB Are Part of the Nitrate Assimilatory Pathway in *Sinorhizobium meliloti*

The denitrification pathway including the nitrate reductase Nap and the nitrite reductase NirK has been described in different rhizobia and especially in *B. japonicum* and *S. meliloti* (Figure 1; Torres et al., 2011, 2014). Surprisingly the nitrate assimilation pathway in *S. meliloti* has received less attention. Eventhough *nirBD* and *narB* were suggested to be part of this pathway and biochemical data obtained from crude extracts of *S. meliloti* indicated the existence of two kinds of nitrate reductase activities (assimilatory and dissimilatory) (Sekiguchi and Maruyama, 1988; Ferroni et al., 2011; Luque-Almagro et al., 2011; Kumar Halder and Chakrabartty, 2015), experimental data were really scarce on the whole. We initiated a study to determine whether NarB and NirB are part of the nitrate assimilatory pathway.

*narB* and *nirB* display an expression pattern different from that of denitrification genes. Indeed both genes are expressed under microaerobic and aerobic conditions, which is not the case for denitrification genes. Strikingly, although their expression level was measurable when cells were grown in the presence of NH_4_Cl, it was highly induced (∼1000-fold) when NH_4_Cl was removed from the medium. In previous work, assimilatory nitrate and nitrite reductase activities were observed only in bacteria grown with nitrate as sole nitrogen source and thus they were described to be inducible (Kumar Halder and Chakrabartty, 2015). In that work, nitrite levels were measured in the medium of cultures grown in presence of glutamate as nitrogen source and found to be inhibited by NH_4_Cl. Our results show that this regulation occurs at the transcriptional level.

RT-PCR experiments demonstrated that *nirB, nirD*, *narB* and *cysG*, the gene located downstream of *narB*, constitute a single transcriptional unit. *cysG* encodes a putative uroporphyrin-III C-methyltransferase involved in the synthesis of sirohaem, the nitrite reductase cofactor (Luque-Almagro et al., 2011). In all conditions tested, *nirB* and *narB* displayed similar expression profiles confirming that they belong to a same operon.

**FIGURE 8:**
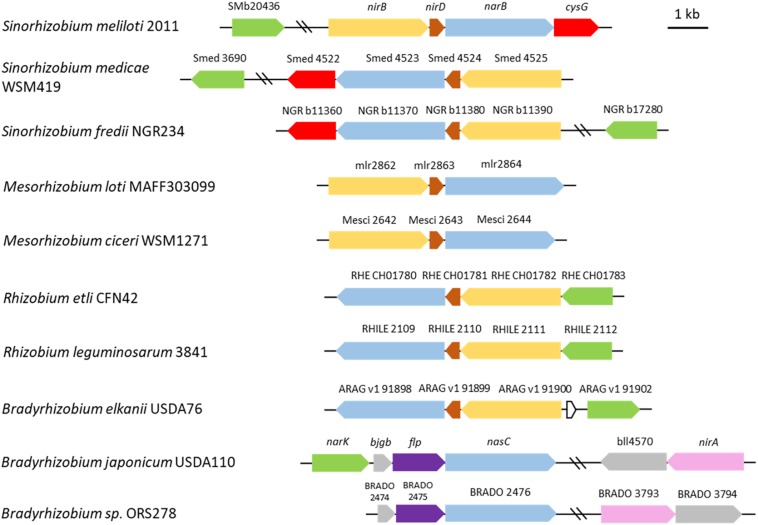
Genomic arrangement of genes involved in nitrate assimilation in selected rhizobia. Genes encoding the assimilatory nitrate reductase (NarB) and nitrite reductase (NirB) in *Sinorhizobium meliloti* are colored in blue and yellow, respectively. Other genes are colored as follow: brown, *nirD* (encoding a small nitrite reductase subunit); green, SMb20436 (encoding the putative nitrite transporter); red, *cysG* (SMb20987) (encoding the putative protein involved in the synthesis of sirohaem, the nitrite reductase cofactor); purple, *flp*. Closest homologs (protein identity) found in other rhizobia are shown with the same color code. The assimilatory nitrite reductase NirA (pink) in *Bradyrhizobium japonicum* doesn’t display significant identity with NirB. The scheme was constructed based on data from the Microbial Genome Annotation & Analysis Platform MAGE (http://www.genoscope.cns.fr) database. The strains are listed according to their phylogenetic proximity from *S. meliloti.*

It is interesting to note that synteny of these genes is maintained in about one third of the alpha-proteobacteria reference genomes (214) and in half of the 25 rhizobial reference genomes found in the Microbial Genome Annotation & Analysis Platform MAGE^1^ database. This organization is not conserved in *B. japonicum* where the assimilatory nitrite reductase encoded by *nirA* is located at a locus distinct from *nasC* (Figure 8). *nirD* encodes a putative protein which displays homology with a nitrite reductase small subunit and is conserved among rhizobia (Figure 8) except most Bradyrhizobial reference genomes (MAGE).

The regulation mechanism of these genes is not known yet in *S. meliloti*. In *B. japonicum* NasT and NasS are involved in the regulation of the assimilatory nitrate reductase encoding gene *nasC* and *nirA*, NasS being a nitrate/nitrite sensor and NasT predicted to be a transcription anti-terminator (Luque-Almagro et al., 2011; Cabrera et al., 2016). *nasTS* are located close to *nirA* on the *B. japonicum* genome and this architecture is conserved in about 57% of reference bradyrhizobial genomes (MAGE) indicating that NasST could act at distance or that they might also be involved in the regulation of other genes or metabolic pathways. *S. meliloti* encodes homologs of NasS and NasT, SMb21114 (46.8% identity) and SMb21115 (65.4% identity), respectively. It will be interesting to test whether these genes are involved in regulation of nitrate assimilation. Interestingly, microarray experiments analyzing the transcriptome of *S. meliloti* cells entering the stationary phase following a nitrogen deprivation showed that *narB* and *nirB* are induced about 15-fold together with SMb21114 and SMb21115 (12- and 65-fold, respectively) (Sauviac et al., 2007, LS and CB, unpublished). In addition, a small non-coding RNA (160 bp) has been annotated on the antisense strand of the *narB* genomic sequence (SMb23331). Interestingly we have observed that in the different zones of the nodule, the expression pattern of this ncRNA is inversely correlated to that of *narB* and *nirB* (Roux et al., 2014). Work is underway to determine the role of SMb21114/21115 and ncRNA SMb23331 on *narB nirB* expression.

Finally, a *S. meliloti* strain deleted for *narB* and *nirBD* failed to grow in a medium with nitrate as the sole nitrogen source. On the whole our results support the conclusion that NarB and NirBD are involved in the nitrate assimilatory pathway in *S. meliloti*.

### NarB Contributes to the Production of NO via the Denitrification Pathway in Free-Living *S. meliloti* Cells

Nitric oxide is an important gas signaling molecule which has a role in diverse biological processes in eukaryotic as well as prokaryotic cells. NO is produced by mammalian cells in response to pathogen attack but surprisingly this molecule is also produced by the pathogen itself. Indeed some gram positive pathogens including *Bacillus* or *Staphylococcus* species were shown to possess bacterial analogs of mammalian NO synthases (Crane et al., 2010).

Surprisingly in the legume-rhizobium symbiotic interaction, NO is also produced by both partners (Sánchez et al., 2010; Horchani et al., 2011). The proportion of NO produced by the bacteria is variable depending upon the legume-rhizobium model considered. Indeed in soybean nodules about 90% of the NO detected is produced by the symbiont *B. japonicum* (Sánchez et al., 2010). These data were confirmed very recently by means of a fluorescent probe as well as electron paramagnetic resonance spectroscopy to assess NO production in nodules (Calvo-Begueria et al., 2018). On the other hand 35% of the NO found in *M. truncatula* nodules is produced by *S. meliloti*. In both cases the role of NO produced by the bacterial partner is still puzzling in the symbiotic context. *S. meliloti* does not contain any NO synthase gene in its genome. Instead, NO synthesized by rhizobium species is thought to be an intermediate product of denitrification. In this process bacteria produce NO as an intermediate of nitrate reduction to N_2_ with the purpose of acquiring energy (“nitrate respiration”) or balancing the redox state during oxygen deprivation conditions. The fraction of NO produced by *S. meliloti* in *M. truncatula* nodules has been estimated by using bacterial strains affected in the denitrification genes *napA* and *nirK*. This amount could have been underestimated if other sources of NO exist in the bacteria. In *B. japonicum*, it was shown that NasC is involved in nitrate assimilation but also in NO production (Cabrera et al., 2016). In the present study we show that a *S. meliloti* mutant strain affected in the periplasmic nitrate reductase NapA still produces NO although at a lower level (39% of the WT), suggesting that there is another way to produce NO from nitrate in *S. meliloti* cells. Indeed our data indicate that NarB participates to NO synthesis as the production of NO was diminished by 80% as compared to the WT strain when *narB* was deleted. Interestingly, NO was barely detectable when measured in a WT strain under conditions where denitrification is not active. This confirms that the assimilatory nitrate reductase contributes to NO production indirectly via the denitrification pathway. NO_2_^–^ produced in the cytoplasm by NarB could serve as substrate for NirK in the periplasm. To render this possible, nitrite must be exported to the cell periplasm. In *B. japonicum* NarK might insure this role and it is interesting to note that the closest NarK homolog in *S. meliloti* (26.5% identity) is encoded by SMb20436 which has not been characterized yet but was found to be highly induced under nitrogen deprivation conditions (LS and CB, unpublished). Its involvement in NO_2_^–^ export remains, however, to be examined. Our results confirm that denitrification remains the main source of NO in *S. meliloti* as NO production was almost null when *nirK* was mutated indicating also that there is no other detectable source of NO in these conditions. NarB contributes to NO production via the denitrification pathway even in presence of ammonium (data not shown) and enhances greatly this production in condition where the *narB* gene is fully expressed. Whether this synergy has a role to play in *S. meliloti* metabolism is a question which remains to be addressed.

### NarB and NirB Do Not Play a Major Role in the Symbiotic Interaction

NarB and NirB are part of the *S. meliloti* nitrate assimilatory pathway, and contribute to NO production in free living cells. Both genes are specifically expressed in the nitrogen fixing zone of *M. truncatula* nodules. This might seem in contradiction with the fact that ammonium produced from nitrogen fixation is present in nodules. However, the assimilation rate of ammonium by the plant might be high enough to avoid any accumulation of ammonium in nodules. Results which might appear contradictory have been published in the past concerning the link between nitrogen fixation and nitrate reduction: indeed a significant correlation between nitrate reductase and nitrogenase activities was suggested in *S. meliloti* (Kondorosi et al., 1973) while no correlation between nitrate reductase (including assimilatory nitrate reductase) and nitrogenase activities was found in a different study (Antoun et al., 1980). Recently Liu and colleagues have shown, by using three *Sinorhizobium* species, a lineage-dependent contribution of the *nap nir* gene cluster to the symbiosis efficiency with soybean (Liu et al., 2017). Here we found that inoculating *M. truncatula* with *S. meliloti* strains affected in *narB*, or *nirB*-*narB* region do not affect nodulation kinetics or plant shoot dry weight or nitrogen fixation efficiency suggesting that these genes do not play an essential role in symbiosis. The questions remain whether these genes participate to NO production in nodules and what their function in symbiosis could be.

## Author Contributions

BR carried out the experiments. ALS and AR constructed the *narB* and *nirBD narB* mutants. LS supervised BR, ALS, and AR works. CB and EM supervised the project and wrote the manuscript.

## Conflict of Interest Statement

The authors declare that the research was conducted in the absence of any commercial or financial relationships that could be construed as a potential conflict of interest.

## Footnotes

1 http://www.genoscope.cns.fr
